# Tubular epithelial cell-derived extracellular vesicles induce macrophage glycolysis by stabilizing HIF-1α in diabetic kidney disease

**DOI:** 10.1186/s10020-022-00525-1

**Published:** 2022-08-12

**Authors:** Yijie Jia, Jiaqi Chen, Zhikang Zheng, Yuan Tao, Shuting Zhang, Meina Zou, Yanlin Yang, Meng Xue, Fang Hu, Yang Li, Qian Zhang, Yaoming Xue, Zongji Zheng

**Affiliations:** 1 Department of Endocrinology & Metabolism, Nanfang Hospital, Southern Medical University, Guangzhou, 510515 China; 2 The First School of Clinical Medicine, Southern Medical University, Guangzhou, China; 3 Department of Endocrinology, Guangdong General Hospital/Guangdong Academy of Medical Sciences, Guangzhou, China; 4 Department of Endocrinology and Metabolism, Shenzhen People’s Hospital (The Second Clinical Medical College, The First Affiliated Hospital, Southern University of Science and Technology), Jinan University, Shenzhen, China; 5 Department of Endocrinology and Metabolism, The Fifth Affiliated Hospital Sun Yat-Sen University, Zhuhai, Guangdong China; 6 Department of Geriatrics, Zhujiang Hospital, Southern Medical University, Guangzhou, China

**Keywords:** Diabetic kidney disease, Extracellular vesicles, Macrophage, Glycolysis

## Abstract

**Background:**

Albuminuria is a hallmark of diabetic kidney disease (DKD) that promotes its progression, leading to renal fibrosis. Renal macrophage function is complex and influenced by macrophage metabolic status. However, the metabolic state of diabetic renal macrophages and the impact of albuminuria on the macrophage metabolic state are poorly understood.

**Methods:**

Extracellular vesicles (EVs) from tubular epithelial cells (HK-2) were evaluated using transmission electron microscopy, nanoparticle tracking analysis and western blotting. Glycolytic enzyme expression in macrophages co-cultured with HSA-treated HK-2 cell-derived EVs was detected using RT-qPCR and western blotting. The potential role of EV-associated HIF-1α in the mediation of glycolysis was explored in HIF-1α siRNA pre-transfected macrophages co-cultured with HSA-treated HK-2 cell-derived EVs, and the extent of HIF-1α hydroxylation was measured using western blotting. Additionally, we injected db/db mice with EVs via the caudal vein twice a week for 4 weeks. Renal macrophages were isolated using CD11b microbeads, and immunohistofluorescence was applied to confirm the levels of glycolytic enzymes and HIF-1α in these macrophages.

**Results:**

Glycolysis was activated in diabetic renal macrophages after co-culture with HSA-treated HK-2 cells. Moreover, HSA-treated HK-2 cell-derived EVs promoted macrophage glycolysis both in vivo and in vitro. Inhibition of glycolysis activation in macrophages using the glycolysis inhibitor 2-DG decreased the expression of both inflammatory and fibrotic genes. Mechanistically, EVs from HSA-stimulated HK-2 cells were found to accelerate macrophage glycolysis by stabilizing HIF-1α. We also found that several miRNAs and lncRNAs, which have been reported to stabilize HIF-1α expression, were increased in HSA-treated HK-2 cell-derived EVs.

**Conclusion:**

Our study suggested that albuminuria induced renal macrophage glycolysis through tubular epithelial cell-derived EVs by stabilizing HIF-1α, indicating that regulation of macrophage glycolysis may offer a new treatment strategy for DKD patients, especially those with macroalbuminuria.

**Supplementary Information:**

The online version contains supplementary material available at 10.1186/s10020-022-00525-1.

## Background

Albuminuria is an independent risk factor for the development of diabetic kidney disease (DKD) and can directly cause renal injury (Skupien et al. 2012; Coresh et al. 2019). Several studies have found that reducing albuminuria could delay the development of end-stage renal disease (Zeeuw 2007; Weir 2015). However, these strategies remain unsatisfactory, especially for individuals with macroalbuminuria.

Tubular epithelial cells, which reabsorb excessive urinary albumin, can release chemokines to induce immune cells, leading to renal injury (Morii et al. 2003). Macrophages, the most important type of immune cell in renal tissue, act as inducers of renal fibrosis (Wen and Crowley 2020). The function of macrophages in the progression of DKD is complex. Most studies have found that macrophages play their role via polarization to the M1 phenotype and by releasing inflammatory mediators (You et al. 2013). Some studies have also found that renal macrophages express fibrotic genes (Landis et al. 2018; Calle and Hotter 2020). Notably, the abovementioned functions can lead to renal fibrosis. Because macrophages play a complex role during disease progression (Mosser and Edwards 2008), further exploration of the mechanism underlying their role in DKD is needed.

Studies have shown that metabolic changes in macrophages under injury stimulation are similar to those in tumor cells (Riksen and Netea 2021). After stimulation, macrophages cause substantial activation of the glycolytic pathway, which is characterized by increased glucose consumption, lactic acid synthesis and glycolysis. Such changes can quickly provide energy to macrophages and promote rapid cell proliferation (Kasmi and Stenmark 2015). Recent studies have shown that both phenotypes of macrophages exhibit increased glycolytic activation (Langston et al. 2017). Moreover, overexpression of glycolytic enzymes can promote macrophages to release factors and glycolysis products, which directly contribute to tissue fibrosis (Liu and Xu 2020).

Recent studies have found that crosstalk between tubular epithelial cells and macrophages is key in regulating macrophage function (Kooten and Daha 2001; Bolisetty et al. 2015; Masola et al. 2018). Emerging evidence indicates that extracellular vesicles (EVs) mediate the transfer of information from tubular epithelial cells to macrophages (Lv et al. 2018; Jia et al. 2019; Jiang et al. 2022). EVs are small membrane particles secreted by all types of cells that can carry messages via molecules such as nucleic acids and proteins to recipient cells (Colombo et al. 2014). We have previously found that albumin could promote macrophage M1 polarization through EVs (Jia et al. 2019). Thus, we proposed that tubular epithelial cell-derived EVs may also influence the macrophage metabolic state.

The metabolic state of renal macrophages during DKD is still unclear. Zeng et al. found that high glucose could upregulate bone marrow-derived macrophage glycolysis (Zeng et al. 2020). However, whether the albumin that is reabsorbed by tubular epithelial cells can affect the metabolic state of macrophages through EVs is unknown. Here, we investigated the influence of albumin on the metabolic state of renal macrophages during DKD and the underlying mechanism.

## Methods

### Mice

All animals were housed under specific pathogen-free conditions, and experiments were administered according to the guidelines of the Institutional Animal Care and Use Committee of the Laboratory Animal Center at Southern Medical University and in accordance with the NIH guidelines for the Care and Use of Laboratory Animals (certificate number: L2018022). Type 2 diabetes mellitus model db/db mice (male, n = 15) and age-matched nondiabetic littermates db/m mice (male, n = 5) on the C57BLKS/J background were purchased at 9 weeks of age from the Model Animal Research Center of Nanjing University and sacrificed at 24 weeks. To study the effects of EVs, ten db/db mice were randomly divided into two groups: the db/db + Control-HK-2-EVs group (n = 5) injected with HK-2 cell-derived EVs (100 µg) via the caudal vein twice a week for 4 weeks, and the db/db + HSA-HK-2-EVs group (n = 5) injected with HSA-treated HK-2 cell-derived EVs (100 µg).

### Macrophage isolation from mouse kidneys

Mouse kidney samples were isolated and digested in DMEM with 1 mg/L collagenase for 1 h and subsequently filtered through a 40-µm mesh. The filtered suspension was isolated using CD11b microbeads (130-093-634; Miltenyi Biotec) according to the manufacturer’s instructions to obtain macrophages.

### Cell culture

The human tubular epithelial cell line HK-2 and the human myeloid leukemia mononuclear cell line THP-1 were obtained from the Cell Bank of the Type Culture Collection (Chinese Academy of Sciences, Shanghai, China) and maintained in Roswell Park Memorial Institute 1640 medium containing 10% fetal bovine serum (FBS) (Gibco, Australia) at 37 °C under a 5% CO_2_ atmosphere.

For treatment with human serum albumin (HSA), HK-2 cells were cultured in RPMI 1640 medium containing 2% FBS for 24 h and then stimulated with 20 mg/ml HSA for 48 h. For lipopolysaccharide (LPS) stimulation, THP-1 cells were induced to macrophages using 100 nM phorbol myristate acetate (PMA) (S1819; Beyotime, China) for 48 h and treated with 100 ng/ml LPS (S1732; Beyotime) for another 24 h. For investigations with the prolyl hydroxylase inhibitor FG-4592, macrophages were treated with 5 mM FG-4592 (SC1135; Beyotime) for 24 h.

HK-2 cells and macrophages were transfected using Lipofectamine 3000 (Invitrogen, Carlsbad, USA). To knock down Rab27a, HK-2 cells were transfected with 50 nM Rab27a siRNA (RiboBio, Guangzhou, China). For HIF-1α knockdown, macrophages were transfected with 50 nM HIF-1α siRNA (RiboBio, Guangzhou, China). Transwell co-culture systems were applied for co-culture with HK-2 cells and macrophages (Corning, MA, USA).

### EV isolation, preparation, and identification

For control group HK-2 cell-derived EVs (Control-HK-2-EVs) isolation, the HK-2 cells were changed to substrate with 2% EV-depleted FBS when the cells reached 60% confluence, and after 48 h, the medium was collected. For HSA-treated HK-2 cell-derived EVs (HSA-HK-2-EVs) isolation, HK-2 cells were treated with HSA for 24 h, washed twice with PBS and the medium was changed to substrate with 2% EV-depleted FBS for 48 h of culture. The culture media was then centrifuged at 3000×*g* for 15 min at 4 °C to remove cellular debris. Next, a 1/5 proportion of ExoQuick‐TC (System Biosciences, USA) was applied, and the compound was hatched overnight at 4 °C. The following day, the mixture was spined at 1300×*g* for 30 min, and then the pellet was dissolved in PBS and purified through a 0.22-μm filter. The EV morphology was examined as follows. First, fresh pellets were loaded onto 200-mesh nickel grids for 1 min, and then one drop of 2% phosphotungstic acid was added for 1 min of incubation. After the phosphotungstic acid was removed, the pellets were allowed to air dry. The shape and size of the EVs were determined using a transmission electron microscope (Hitachi H-7650, Japan). EVs were diluted using 1 ml of 1× PBS buffer, and the initial diluted sample was fed into the sample tank through a syringe. The particle size and concentration were measured using a NanoSight NS300 (Malvern, UK).

### Co-culture of macrophages with EVs

Macrophages were treated with 30 µg/ml HK-2 cell-derived EVs for 24 h. To assess EVs uptake by macrophages, we labeled HK-2 cell-derived EVs using Dil-C18 (5 µl/ml) and cultivated them at 37 °C for 30 min. The unbound dye was removed with exosome spin columns (MW 3000, Invitrogen). Then, macrophages were co-cultured with the labeled EVs for 24 h and stained with DAPI. Images were captured using an Olympus microscope (Japan).

### In vivo biodistribution of EVs in mice

To assess the biodistribution of the EVs in mice, we used 5 µl/ml DiD (KGMP0025, KeyGEN BioTECH, China)-labeled HK-2 cell-derived EVs, and the unbound dye was removed with exosome spin columns (MW3000, Invitrogen). Then, 100 µg of HK-2 cell-derived EVs was injected into C57BL/6J mice (8 weeks old) via the caudal vein. After 24 h, the tissue samples were harvested and observed using an in vivo imaging system (IVIS) spectrum (Ami HTX, Spectral Instruments Imaging, USA).

### Western blot analysis

The frozen renal cortex samples were ground by a Lu Ka sample freezing grinder (LUKYM-I, Guangzhou). Equal amounts of protein were separated by SDS-PAGE and transferred to PVDF membranes. Then, the membranes were incubated with 10% BSA in Tris/Tween-buffered saline and primary antibodies against LDHA (19987-1-AP; Proteintech, USA), HK2 (22029-1-AP; Proteintech, USA), HIF-1α (20960-1-AP; Proteintech, USA), CD63 (A5271; ABclonal, USA), TSG101 (A2216; ABclonal, USA), and β-actin (FD0060; Fdbio Science, China) overnight. Bands were detected with an ECL Plus western blotting detection system (GelView 6000 Pro, Guangzhou, China) and analyzed with Quantity One software (Bio-Rad, CA, USA).

### qRT-PCR

TRIzol reagent (Invitrogen) was used to extract total RNA from renal tissue or cells. The Hieff First-Strand cDNA Synthesis Super Mix for RT-qPCR kit (Yeasen Biotech, Shanghai) was used for reverse transcription. The Hieff qPCR SYBR Green Master Mix kit (Yeasen Biotech, Shanghai) and a LightCycler 480 (Roche, Basel, Switzerland) were used for real-time PCR. Expression levels were calculated relative to the expression of β-actin. The primer sequences are shown in Additional file 1: Table S1.

### Lactate measurements

The levels of lactate in the macrophage supernatants were measured using a lactate assay kit (KGT023, KeyGEN BioTECH, China) following the manufacturer’s instructions.

### Immunohistochemistry

Kidney samples were cut into 4-µm sections. The samples were stained with periodic acid-Schiff (PAS) for histological analysis. Other slides were incubated with primary antibodies against FN (15613-1-AP, Proteintech, Rosemont, USA), α-SMA (ab5694, Abcam, Cambridge, MA), Col-I (ab270993, Abcam) and IL-6 (66146-1-Ig, Proteintech) overnight at 4 °C and then incubated with secondary antibodies. Finally, an Olympus B upright light microscope was used for image capture (Olympus, Japan). The sizes of the stained areas were calculated by ImageJ software. For quantification, each tissue section was divided into four quadrants, and four fields were randomly selected from each quadrant for further analysis.

### Immunohistofluorescence analyses

Paraffin sections from mouse kidneys were used for immunohistofluorescence analyses. After antigen repair, the kidney sections were blocked with 10% normal goat serum for 30 min and then incubated with primary antibodies against F4/80 (clone BM8, eBioscience, USA) at 4 °C overnight to stain macrophages. Then, the kidney sections were incubated with anti-GLUT1 (21829-1-AP, Proteintech, USA) and anti-HIF-1α (20960-1-AP; Proteintech, USA) antibodies at 4 °C overnight followed by incubation with secondary antibodies. DAPI was used for nuclear staining. A Zeiss epifluorescence microscope was used to capture images.

### Statistical analyses

Data are presented as the mean ± SEM, and n indicates the number of animals or number of assays performed. One-way ANOVA was employed for comparisons between multiple groups. Student’s t test was used for two-group comparisons. Differences for which the *p* value was < 0.05 were considered statistically significant.

## Results

### Glycolysis was increased in diabetic kidney macrophages

**Fig. 1:**
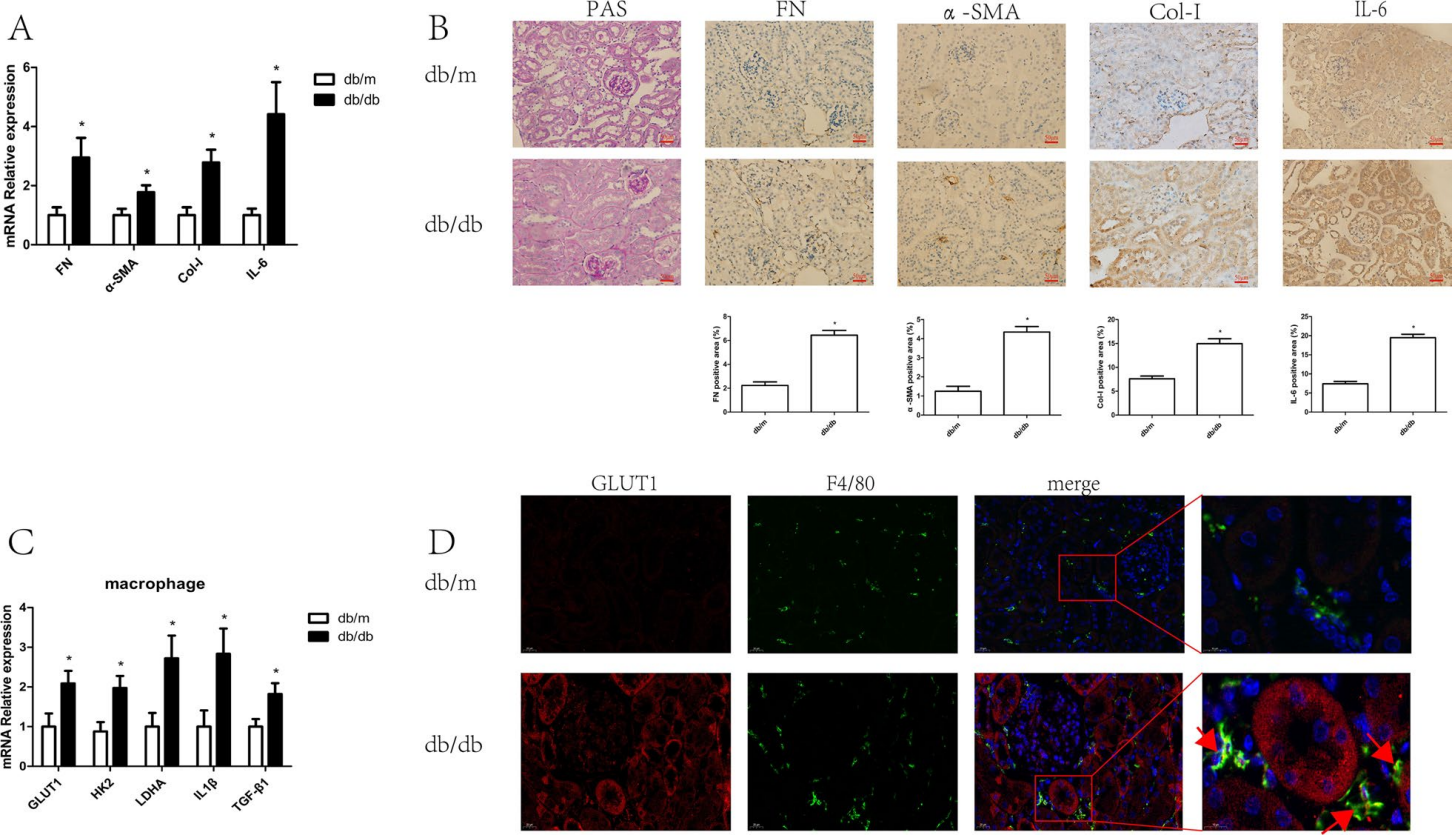
Glycolysis increased in diabetic kidney macrophages. **A** The mRNA expression of FN, α-SMA, Col-I and IL-6 in the kidney cortexes of db/m (n = 5) and db/db (n = 5) mice. **B** PAS staining and immunostaining of FN, α-SMA, Col-I and IL-6 (original magnification ×400) in the kidney cortexes of db/m (n = 5) and db/db (n = 5) mice. **C** mRNA expression of GLUT1, HK2, LDHA, IL1β and TGF-β1 in the kidney macrophages of db/m (n = 5) and db/db (n = 5) mice. **D** Double immunohistochemistry (IHC) staining of GLUT1 (red) and F4/80 (green) in the kidney cortexes of db/m and db/db mice. *p < 0.05 vs. db/m group

To explore the metabolic state of renal macrophages during DKD, we used db/db mice, which, in comparison with db/m mice, show enhanced expression of both fibrosis (FN, α-SMA and Col-I) and inflammation (IL-6) markers by qRT-PCR (Fig. 1A) and immunohistochemical staining (Fig. 1B). To explore the metabolic state and function of renal macrophages during DKD, we isolated mouse renal macrophages and found significant increases in GLUT1, HK2 and LDHA mRNA expression in db/db mice in contrast with that in db/m mice (Fig. 1C). Consistent with the qRT-PCR findings, immunofluorescence indicated that GLUT1 levels were much higher in db/db mouse kidney F4/80 macrophages (Fig. 1D). Furthermore, we found that IL1β and TGF-β1 mRNA levels were increased in renal macrophages from db/db mice (Fig. 1C). Collectively, these facts suggested that glycolysis was enhanced in DKD mouse renal macrophages.

### HSA-treated tubular epithelial cells promoted macrophage glycolysis

**Fig. 2:**
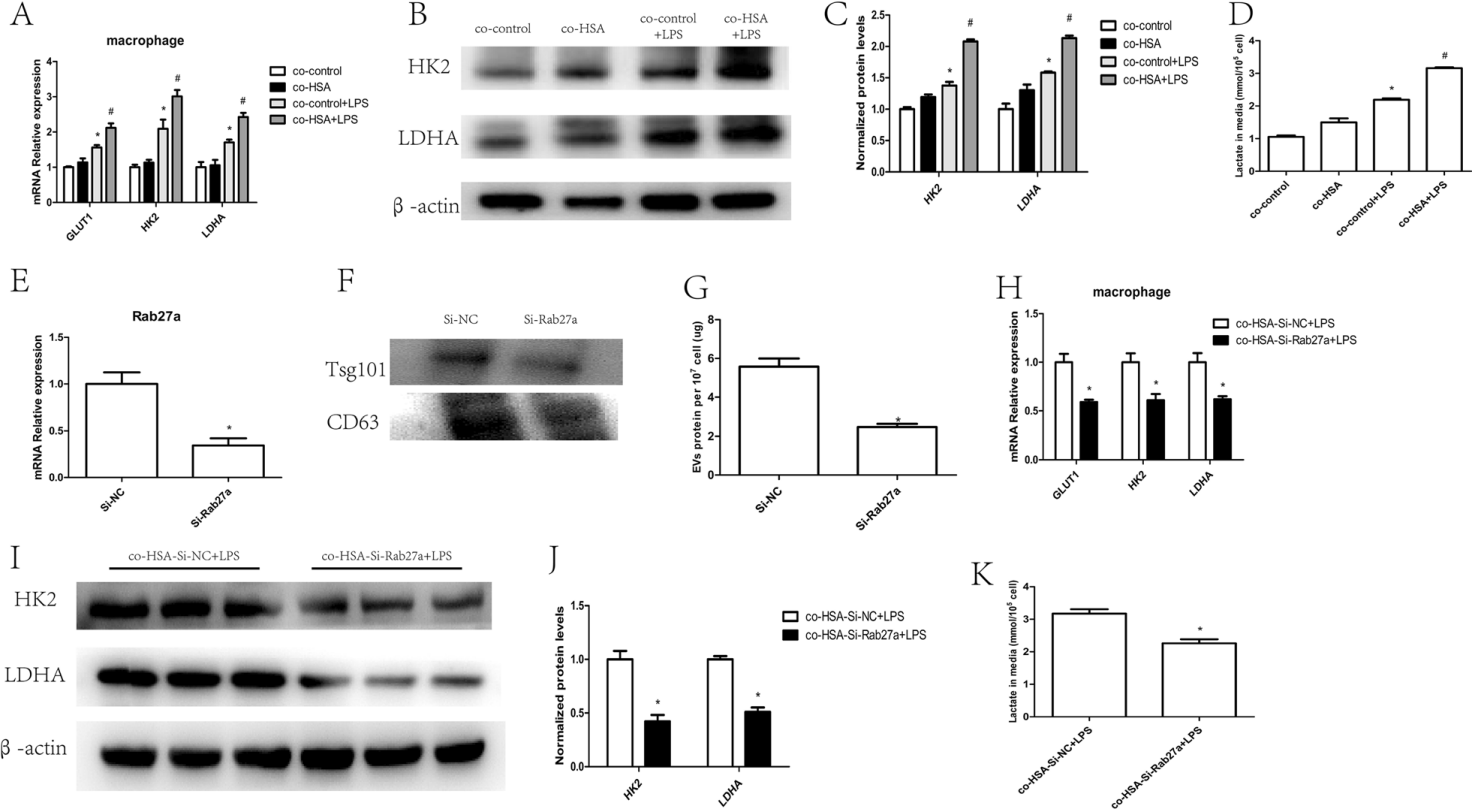
HSA-treated tubular epithelial cells promoted macrophage glycolysis. Macrophages were co-cultured with HK-2 cells. **A** mRNA expression of GLUT1, HK2 and LDHA; **B**, **C** protein levels of HK2 and LDHA (n = 3); **D** amount of lactate in the macrophage supernatant (n = 3); *p < 0.05 vs. the co-control group; #*p* < 0.05 vs. the co-control + LPS group. HK-2 cells were transfected with Rab27a siRNA: **E** mRNA levels of Rab27a in HK-2 cells (n = 3); **F** EV protein levels of CD63 and Tsg101 from the same number of HK-2 cells; **G** total EV protein from the same number of tubular epithelial cell-derived EVs (n = 3); *p < 0.05 vs. the Si-NC group. Macrophages were co-cultured with HK-2 cells transfected with Rab27a siRNA or Si-NC: **H** mRNA levels of GLUT1, HK2 and LDHA (n = 3); **I**, **J** protein levels of HK2 and LDHA (n = 3); **K** amount of lactate in the macrophage supernatant (n = 3); *p < 0.05 vs. the co-HSA-Si-NC + LPS group

Proteinuria is key to the progression of DKD, and excess albumin affects macrophages through tubular epithelial cells (Jia et al. 2019). To further explore whether albumin-treated tubular epithelial cells also influence the macrophage metabolic state, we measured the expression of glycolytic enzymes. Macrophage co-culture with HSA-treated HK-2 cells greatly enhanced GLUT1, HK2 and LDHA expression at the mRNA level (Fig. 2A). Changes in the protein expression of HK2 and LDHA paralleled that of their mRNA expression (Fig. 2B, C) in macrophages. We also found markedly higher lactate levels in the macrophage supernatant after co-incubation with HSA-treated HK-2 cells (Fig. 2D).

In our previous research, HSA-treated tubular epithelial cells were found to affect macrophage phenotypes through EVs (Jia et al. 2019). To further explore whether EVs are essential in macrophage glycolysis, we inhibited HK-2 cell-derived EV secretion and then co-cultured these cells with macrophages. Rab27a, a member of the Rab family of small GTPases, plays a key role in EV secretion. We inhibited Rab27a expression using Rab27a siRNA (Fig. 2E). The expression of EV markers was reduced in EVs derived from HK-2 cells that were pretransfected with Rab27a siRNA compared with the same number of non-pretransfected HK-2 cells at the protein level (Fig. 2F). Furthermore, using protein quantification, we found that the number of EVs was significantly decreased in cells transfected with Rab27a siRNA (Fig. 2G). Then, we co-cultured HSA-stimulated and Rab27a siRNA-transfected HK-2 cells with macrophages. Glycolytic enzymes were greatly decreased in macrophages co-incubated with HK-2 cells pretransfected with Rab27a siRNA (Fig. 2H–J). Lactate levels were also lower in the macrophage supernatant after co-culture with HK-2 cells pretransfected with Rab27a siRNA (Fig. 2K). These findings indicated that HSA-stimulated tubular epithelial cells affected macrophage glycolysis through EVs.

### HSA-treated tubular epithelial cell-derived EVs promoted macrophage glycolysis

**Fig. 3:**
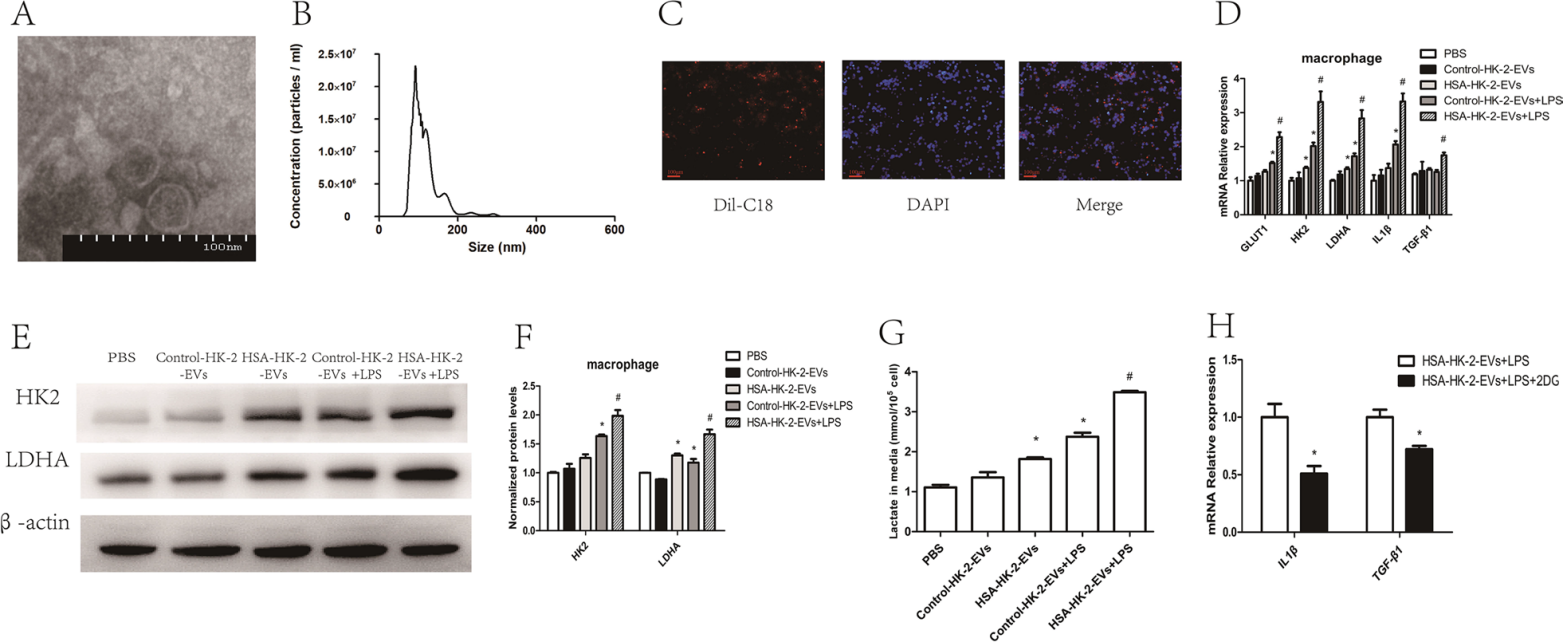
HSA-treated tubular epithelial cell-derived EVs promoted macrophage glycolysis in vitro. **A** EV morphology was analyzed using transmission electron microscopy. **B** EV size distributions were analyzed using nanoparticle tracking analysis. **C** EVs labeled with Dil-C18 were taken up by macrophages (original magnification ×100). Macrophages were co-cultured with EVs: **D** mRNA levels of GLUT1, HK2, LDHA, IL1β and TGF-β1 (n = 3); **E**, **F** protein levels of HK2 and LDHA (n = 3); **G** amount of lactate in the macrophage supernatant (n = 3); *p < 0.05 vs. the Control-HK-2-EVs group; ^#^*p* < 0.05 vs. the Control-HK-2-EVs + LPS group. Macrophages treated with or without 2-DG were co-cultured with HSA-HK-2-EVs: **H** mRNA levels of IL1β and TGF-β1 (n = 3); *p < 0.05 vs. the HSA-HK-2-EVs + LPS group

We further confirmed that tubular epithelial cell-derived EVs influence the macrophage metabolic state. Therefore, we isolated these EVs and characterized their morphology and properties by transmission electron microscopy (Additional file 2: Fig. S1, Fig. 3A) and nanoparticle tracking analysis (Fig. 3B). To explore whether EVs could be internalized by macrophages, we co-cultured Dil-C18-labeled EVs with macrophages. Using immunofluorescence, we found that after 24 h, the EVs (red fluorescence) were colocalized with macrophages (Fig. 3C). Then, we co-incubated macrophages and HSA-treated HK-2 cell-derived EVs and found that glycolytic enzymes were increased in the macrophages (Fig. 3D–F). The macrophage supernatant lactate level (Fig. 3G) and mRNA levels of IL1β and TGF-β1 (Fig. 3D) were also higher in those co-cultured with HSA-treated HK-2 cell-derived EVs. To further confirm that EVs influence macrophage function by promoting macrophage glycolysis, we treated macrophages with the glycolysis inhibitor 2-DG for co-culture with HSA-treated HK-2 cell-derived EVs. As shown in Fig. 3H, 2-DG reversed the upregulation of IL1β and TGF-β1 expression.

**Fig. 4:**
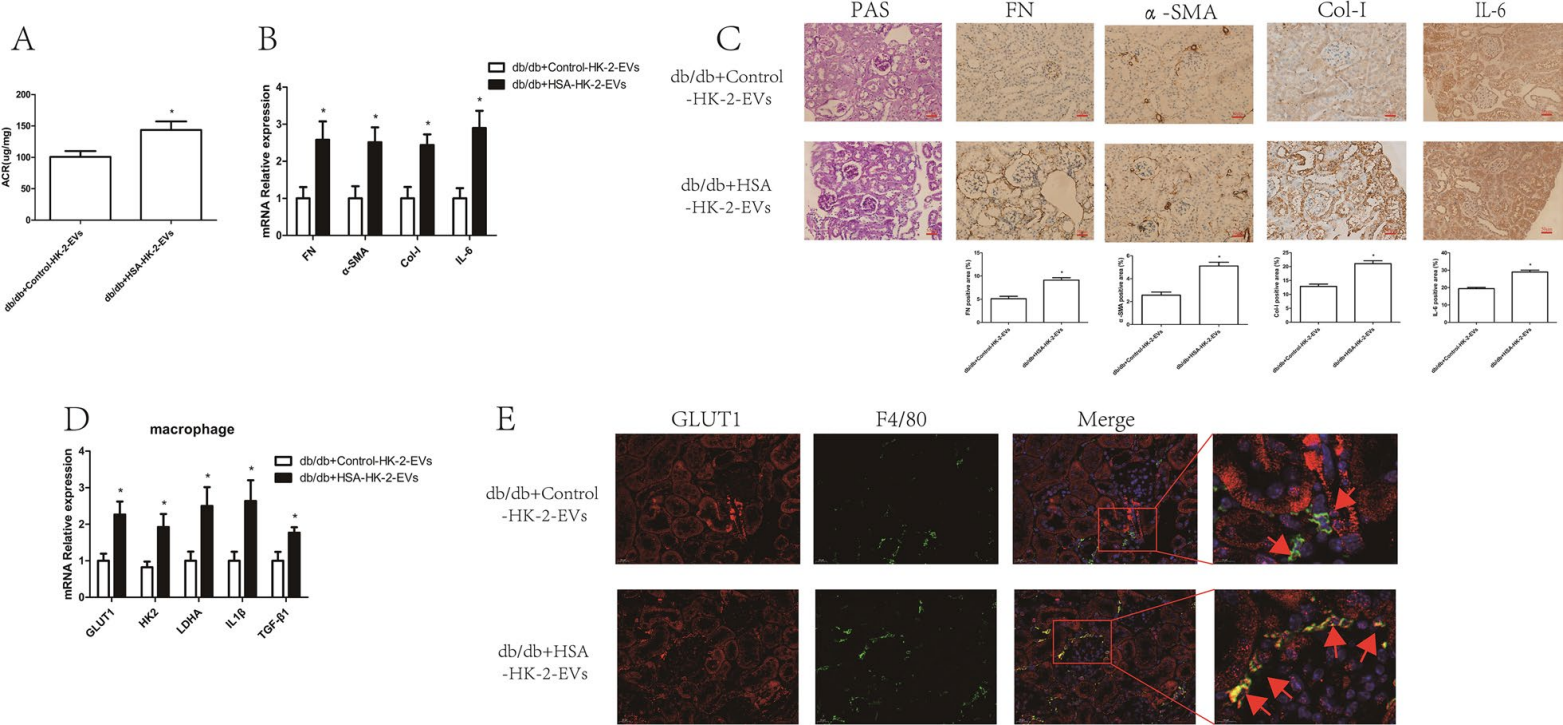
HSA-treated tubular epithelial cell-derived EVs promoted macrophage glycolysis in vivo. db/db mice were injected with Control-HK-2-EVs and HSA-HK-2-EVs via the caudal vein: **A** urinary albumin creatinine ratios (ACRs) of db/db + Control-HK-2-EVs (n = 5) and db/db + HSA-HK-2-EVs (n = 5) mice; **B** mRNA expression of FN, α-SMA, Col-I and IL-6 in the kidney cortexes of db/db + Control-HK-2-EVs (n = 5) and db/db + HSA-HK-2-EVs (n = 5) mice; **C** PAS staining and immunostaining of FN, α-SMA, Col-I and IL-6 (original magnification ×400) in the kidney cortexes of db/db + Control-HK-2-EVs (n = 5) and db/db + HSA-HK-2-EVs (n = 5) mice; **D** mRNA expression of GLUT1, HK2, LDHA, IL1β and TGF-β1 in kidney macrophages of db/db + Control-HK-2-EVs (n = 5) and db/db + HSA-HK-2-EVs (n = 5) mice; **E** double IHC staining of GLUT1 (red) and F4/80 (green) in the kidney cortex; *p < 0.05 vs. the db/db + Control-HK-2-EVs group

To confirm that HSA-treated HK-2 cells derived EVs affect macrophage glycolysis in vivo, we injected EVs into mice through the caudal vein. As shown in Additional file 3: Fig. S2, the majority of EVs appeared in the liver and kidney at 24 h after injection. Furthermore, db/db mice injected with HSA-treated HK-2 cell-derived EVs showed increased levels of ACR (Fig. 4A) and expression of markers of both fibrosis and inflammation in the renal cortex (Fig. 4B, C) and increased glycolysis in renal macrophages (Fig. 4D, E). Taken together, these results indicated that tubular epithelial cell-derived EVs could influence macrophage glycolysis both in vitro and in vivo.

### HIF-1α promoted macrophage glycolysis

**Fig. 5:**
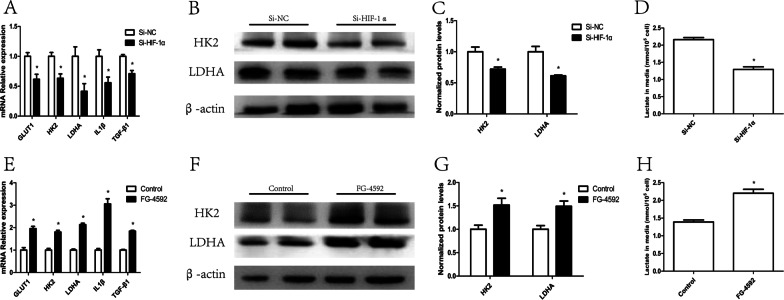
HIF-1α promoted macrophage glycolysis. Macrophages were transfected with HIF-1α siRNA: **A** mRNA levels of GLUT1, HK2, LDHA, IL1β and TGF-β1 (n = 3); **B**, **C** protein levels of HK2 and LDHA (n = 4); **D** amount of lactate in the macrophage supernatant (n = 3); *p < 0.05 vs. the Si-NC group. Macrophages were treated with FG-4592: **E** mRNA levels of GLUT1, HK2, LDHA, IL1β and TGF-β1 (n = 3); **F**, **G** protein levels of HK2 and LDHA (n = 4); **H** amount of lactate in the macrophage supernatant (n = 3); *p < 0.05 vs. the Control group

According to several reports, HIF-1α plays an important role in promoting glycolysis (Cheng et al. 2014). Therefore, to examine whether HIF-1α is essential in regulating macrophage metabolic reprogramming, we transfected macrophages with HIF-1α siRNA or processed macrophages with FG-4592, a prolyl hydroxylase inhibitor, to downregulate or overexpress HIF-1α, respectively (Additional file 4: Fig. S3A–D). We found that HIF-1α siRNA significantly downregulated macrophage GLUT1, HK2, and LDHA mRNA (Fig. 5A) and HK2 and LDHA protein (Fig. 5B, C) expression. The lactate content in the supernatant (Fig. 5D) and the mRNA levels of IL1β and TGF-β1 (Fig. 5A) were also decreased in the HIF-1α siRNA group. On the other hand, FG-4592 upregulated GLUT1, HK2, and LDHA mRNA (Fig. 5E) and HK2 and LDHA protein (Fig. 5F, G) expression. The lactate content (Fig. 5H) and levels of IL1β and TGF-β1 were also higher in the FG-4592 group (Fig. 5E). These data revealed that HIF-1α promotes macrophage glycolysis.

### HSA-treated tubular epithelial cell-derived EVs promoted macrophage glycolysis by stabilizing HIF-1α

**Fig. 6:**
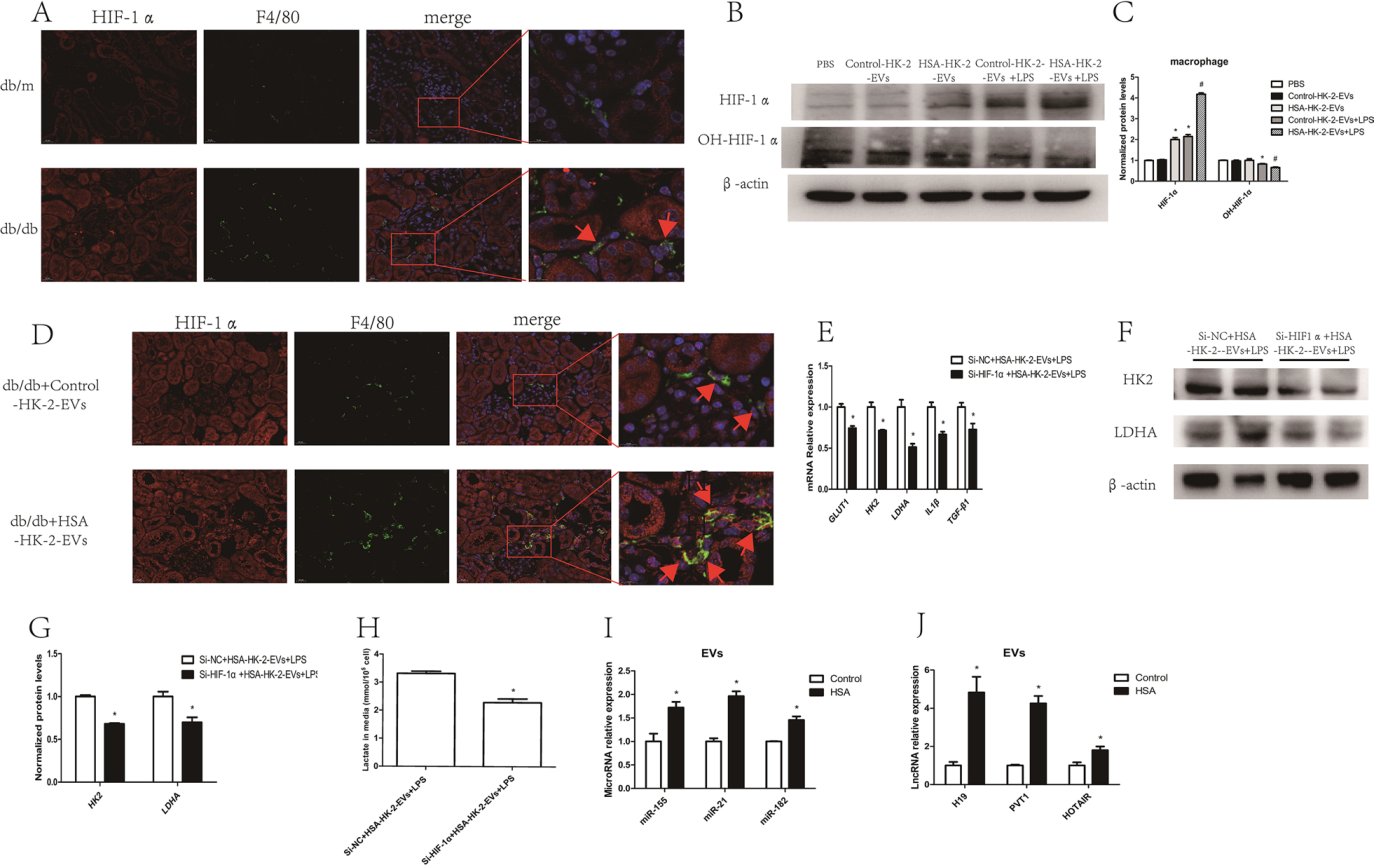
HSA-treated tubular epithelial cell-derived EVs promoted macrophage glycolysis by stabilizing HIF-1α. **A** Double IHC staining of HIF-1α (red) and F4/80 (green) in the kidney cortexes of db/m and db/db mice. Macrophages were co-cultured with tubular epithelial cell-derived EVs: **B**, **C** protein level of HIF-1α and extent of HIF-1α hydroxylation (n = 3); *p < 0.05 vs. the Control-HK-2-EVs group; ^#^*p* < 0.05 vs. the Control-HK-2-EVs + LPS group; **D** double IHC staining of HIF-1α (red) and F4/80 (green) in the kidney cortexes of db/db + Control-HK-2-EVs and db/db + HSA-HK-2-EVs mice. Macrophages transfected with HIF-1α siRNA and co-cultured with HSA-HK-2-EVs: **E** mRNA levels of GLUT1, HK2, LDHA, IL1β and TGF-β1 (n = 3); **F**, **G** protein levels of HK2 and LDHA (n = 4); **H** amount of lactate in the macrophage supernatant (n = 3); *p < 0.05 vs. the Si-NC + HSA-HK-2-EVs + LPS group. MiRNAs and lncRNAs that have been reported to modulate HIF-1α expression were measured in EVs. *p < 0.05 vs. the Control-HK-2-EVs group (n = 3)

To further confirm the role of HIF-1α in HSA-treated tubular epithelial cell-induced macrophage glycolysis, HIF-1α levels in renal macrophages were measured using immunofluorescence. As expected, a markedly higher renal macrophage HIF-1α level was found in db/db mice than in control mice (Fig. 6A). We also found that macrophage HIF-1α expression was increased and the extent of HIF-1α hydroxylation was decreased in cells co-cultured with HSA-treated HK-2 cell-derived EVs (Fig. 5B, C), indicating that HK-2 cell-derived EVs elevate HIF-1α stability. Furthermore, we found increased expression of HIF-1α in the kidney macrophages of db/db mice injected with HSA-treated HK-2 cell-derived EVs (Fig. 6D). To further confirm that HIF-1α participates in glycolysis regulation, we transfected macrophages with HIF-1α siRNA for co-culture with EVs from HSA-stimulated HK-2 cells. As shown in Fig. 6E–H, knockdown of HIF-1α reversed the increases in GLUT1, HK2 and LDHA levels, lactate production, and IL1β and TGF-β1 expression. These results showed that EVs from HSA-stimulated HK-2 cells promoted macrophage glycolysis through HIF-1α. Then, we explored how HSA-treated HK-2 cell-derived EVs regulate HIF-1α stabilization and found that several miRNAs (Jiao et al. 2017; Sun et al. 2020; Li et al. 2015) and lncRNAs (Wang et al. 2020; Guo et al. 2018; Hong et al. 2017) that have been reported to participate in stabilizing HIF-1α were increased in those EVs (Fig. 6I, J).

## Discussion

In this study, we found that renal macrophages from db/db mice showed enhanced glycolysis. Furthermore, EVs derived from HSA-treated tubular epithelial cells promoted renal macrophage glycolysis. Inhibition of macrophage glycolysis activation using 2-DG decreased the expression of both inflammatory and fibrotic genes. Mechanistically, increased HIF-1α stabilization contributed to the induction of macrophage glycolysis induced by HSA-treated renal tubular epithelial cell-derived EVs.

Macrophage accumulation in the kidney is a major feature in the development of DKD and appears at an early stage (Pichler et al. 2017). Additionally, macrophage number is positively correlated with albuminuria and renal fibrosis (Nguyen et al. 2006). Several studies have found that macrophage depletion reduces albuminuria and histological changes in glomeruli (Duffield et al. 2005). Traditionally, macrophages were thought to promote diabetic renal fibrosis through polarization to the M1 phenotype, which leads to inflammation, and several studies have indicated that inhibition of M1 macrophage polarization during DKD could delay renal fibrosis by relieving inflammation. However, some studies have suggested that renal macrophages also express fibrotic genes and directly cause fibrosis, thus playing a role similar to that of M2 macrophages. In a model of renal artery stenosis, macrophages were found to express increased levels of both inflammatory and fibrotic genes (Puranik et al. 2018). In another study, TGF-β expression increased in CD68^+^F4/80^+^ macrophages in the kidneys of mice treated with streptomycin (Cucak et al. 2015). Here, we also found that db/db mice showed increased macrophage expression of IL1β and TGF-β1. Recent studies have discovered that macrophage metabolism and related metabolites are important regulators of macrophage function and phenotype (Saha et al. 2017), but the metabolic state of renal macrophages during DKD is still unclear.

Glycolysis is an important metabolic pathway that can quickly provide energy and support the generation of metabolic products from biosynthetic pathways. M1 macrophages, M2 macrophages and tumor-associated macrophages have been found to exhibit increased glycolysis (Langston et al. 2017; de-Brito et al. 2020), and some studies have shown that the activation of glycolysis increases the levels of inflammatory and fibrotic genes in macrophages (Liu and Xu 2020; Wang and Zhang 2021). Moreover, the products of glycolysis, such as lactic acid and succinate, can also directly cause fibrosis (Kottmann et al. 2012; Ortiz-Masiá and Gisbert-Ferrándiz 2020). In this study, using both qRT-PCR and immunofluorescence, we discovered that glycolysis was enhanced in renal macrophages from db/db mice, which may explain the complex role of macrophages during DKD. Moreover, treatment with 2-DG, an inhibitor of glycolysis, reversed the upregulation of IL1β and TGF-β1 induced by HSA-treated renal tubular epithelial cell-derived EVs. These results indicated that macrophage glycolysis increased during DKD and induced renal fibrosis not only through its products but also the increase in inflammatory and fibrotic gene expression in macrophages.

The metabolic state of macrophages in renal injury has seldom been studied. Jing et al. found that renal macrophages undergo a switch to activate glycolysis in response to IgG IC stimulation, thus contributing to renal inflammation (Jing and Castro-Dopico 2020). In another study, high glucagon levels were thought to be one of the reasons for macrophage glycolysis (Zeng et al. 2020). Albuminuria is one major cause of renal fibrosis during DKD, and whether albuminuria influences macrophage glycolysis, thus promoting renal injury, is unknown. We and others have reported that albuminuria promotes macrophage accumulation and affects macrophage function through renal tubular cell-derived EVs (Lv et al. 2018; Jia et al. 2019). In the present study, we found that macrophage glycolysis induced by HSA intervention in renal tubular cells was reversed by inhibiting renal tubular cell EV secretion. Then, through in vitro and in vivo experiments, we confirmed that HSA enhanced macrophage glycolysis through renal tubular epithelial cell-derived EVs. As albuminuria is an independent risk factor for the development of DKD, our results may provide a new understanding that albuminuria may affect renal macrophage metabolism and promote renal fibrosis.

HIF-1α is a well-known transcription factor that regulates glycolysis-related gene transcription. Several studies have shown that HIF-1α is increased in the kidneys of diabetic (DM) mice, but the function of HIF-1α in the progression of DKD remains controversial (Cai et al. 2020; Jiang et al. 2020; Xie et al. 2019). Here, we found that the HIF-1α level was increased in renal macrophages from db/db mice. After co-culture with HSA-treated HK-2 cell-derived EVs, the extent of HIF-1α hydroxylation decreased, but HIF-1α levels increased, which indicated that HSA-stimulated HK-2 cell-derived EVs could increase the stability of HIF-1α. Through overexpression or downregulation of HIF-1α, HIF-1α regulates glycolytic gene expression in macrophages. To confirm that EVs regulate macrophage glycolysis through HIF-1α, we transfected macrophages with HIF-1α siRNA for co-culture with HSA-treated HK-2 cell-derived EVs, which showed that HIF-1α knockdown reversed the induction of glycolysis. We further preliminarily explored the explanation by which EVs regulate HIF-1α stabilization and found several miRNAs that target PHD2, an enzyme that promotes the hydroxylation and decomposition of HIF-1α, and some lncRNAs that have been reported to elevate HIF-1α stabilization were upregulated in HSA-treated HK-2 cell-derived EVs.

Our study provides novel evidence that albuminuria may affect the metabolic state of renal macrophages through tubular epithelial cell-derived EVs; however, this research still has some limitations. First, many studies have shown that different subpopulations of EVs exert different functions (Tkach and Kowal 2017; Tucher et al. 2018). In our study, functional investigation of EV subpopulations was not possible due to methodological limitations. Moreover, it cannot be ruled out that non-EV components in the separated EV preparations could contribute to the observed results. Therefore, additional studies are needed to assess the functions of different EV subpopulations to regulate the macrophage metabolic state. Second, our study focused on how albumin regulates the macrophage metabolic state. As macrophages have many subtypes and the proportions of these subtypes change during the development of DKD (Calle and Hotter 2020; Zhang et al. 2019), further research may investigate the metabolic state of different subtypes of macrophages during the development of DKD. Third, our study concentrated on only the roles of lncRNAs and miRNAs in EVs, but proteins and other cellular contents also play biological regulatory roles.

## Conclusions

In summary, we found that glycolysis was enhanced in renal macrophages from DM mice. Moreover, HSA promoted macrophage glycolysis through tubular epithelial cell-derived EVs by stabilizing HIF-1α, thus inducing renal fibrosis and inflammation. As albuminuria is an obstacle for DKD treatment, our research indicates that inhibiting renal macrophage glycolysis may be a new method to delay the progression of DKD, especially in individuals with macroalbuminuria.

## Supplementary Information

**Additional file 1: Table S1.** Real time PCR primer sets.

**Additional file 2: Figure S1.** Wide-field image of EV morphology.

**Additional file 3: Figure S2.** In vivo biodistribution of EVs in mice. Imaging of the fluorescence intensity in the mouse organs at 24 h after injection.

**Additional file 4: Figure S3.** HIF-1α expression in macrophages. Macrophages were transfected with HIF-1α siRNA: (A, B) protein levels of HIF-1α (n = 3); *p < 0.05 vs. the Si-NC group. Macrophages were treated with FG-4592: (C, D) protein levels of HIF-1α (n = 3); *p < 0.05 vs. the control group.

## Data Availability

The dataset used and/or analyzed during the current study is available from the corresponding author upon reasonable request.

## Abbreviations

DKD: Diabetic kidney disease
HSA: Human serum albumin
EVs: Extracellular vesicles
ESRD: End-stage renal disease
LPS: Lipopolysaccharide
PMA: Phorbol myristate acetate
HIF-1α: Hypoxia-inducible factor-1 alpha
LDHA: Lactate dehydrogenase A
HK2: Hexokinase 2
GLUT1: Glucose transporter 1
FN: Fibronectin
Col-I: Collagen-I
α-SMA: Alpha-smooth muscle actin
IL-6: Interleukin 6
IL1β: Interleukin-1β
TGF-β1: Transforming growth factor-beta 1
2-DG: 2-Deoxy-d-glucose
FG-4592: Roxadustat
PHD2: Prolyl-4 hydroxylase 2
