# Supplementary material for: Tubular epithelial cell-derived extracellular vesicles induce macrophage glycolysis by stabilizing HIF-1α in diabetic kidney disease
Source: Mol Med. 2022 Aug 12;28:95. doi: 10.1186/s10020-022-00525-1 (PMC9373297; doi:10.1186/s10020-022-00525-1)
Supplement: Supplementary file 1 — Additional file 1: Table S1. Real time PCR primer sets. [file 10020_2022_525_MOESM1_ESM.docx]

| Gene |  | 5’-3’ |
| --- | --- | --- |
| hsa-IL1β | Forward | GAAGCTGATGGCCCTAAACAG |
|  | Reverse | AGCATCTTCCTCAGCTTGTCC |
| hsa-TGF-β1 | Forward | GAAACCCACAACGAAATCTATGAC |
|  | Reverse | ACGTGCTGCTCCACTTTTAACT |
| hsa-HK2 | Forward | GCCCACCTACGTGTGTGCTA |
|  | Reverse | CACCCCACTTCCCATTCCGA |
| hsa-GLUT1 | Forward | ATACTCATGACCATCGCGCTAG |
|  | Reverse | AAAGAAGGCCACAAAGCCAAAG |
| hsa-LDHA | Forward | TAGGCTACAACAGGATTCTAGGTGGAG |
|  | Reverse | GTCAGAGGTGGCAGAACTATTTC |
| hsa-β-actin | Forward | CCCTGGACTTCGAGCAAGAGAT |
|  | Reverse | GTTTTCTGCGCAAGTTAGG |
| mmu-FN | Forward | CGAGGTGACAGAGACCACAA |
|  | Reverse | CTGGAGTCAAGCCAGACACA |
| mmu- Col-1 | Forward | ACATGTTCAGCTTTGTGGACC |
|  | Reverse | TAGGCCATTGTGTATGCAGC |
| mmu-TGF-β1 | Forward | CTTTGTACAACAGCACCCGC |
|  | Reverse | CGGGTGACTTCTTTGGCGTA |
| mmu-αSMA | Forward | TCCCTGGAGAAGAGCTACGAA |
|  | Reverse | ATAGGTGGTTTCGTGGATGCC |
| mmu-IL1β | Forward | TGGTACATCAGCACCTCACA |
|  | Reverse | GAAGGCATTAGAAACAGTCC |
| mmu-GLUT1 | Forward | CAGTTCGGCTATAACACTGGTG |
|  | Reverse | GCCCCCGACAGAGAAGATG |
| mmu-HK2 | Forward | TGATCGCCTGCTTATTCACGG |
|  | Reverse | AACCGCCTAGAAATCTCCAGA |
| mmu-LDHA | Forward | TGTCTCCAGCAAAGACTACTGT |
|  | Reverse | GACTGTACTTGACAATGTTGGGA |
| mmu-β-actin | Forward | CGAGCGTGGCTACAGCTTCA |
|  | Reverse | AGGAAGAGGATGCGGCAGTG |

**Table S1: Real Time PCR Primer Sets**
