# Supplementary figures and images for: Tubular epithelial cell-derived extracellular vesicles induce macrophage glycolysis by stabilizing HIF-1α in diabetic kidney disease
Source: Mol Med. 2022 Aug 12;28:95. doi: 10.1186/s10020-022-00525-1 (PMC9373297; doi:10.1186/s10020-022-00525-1)

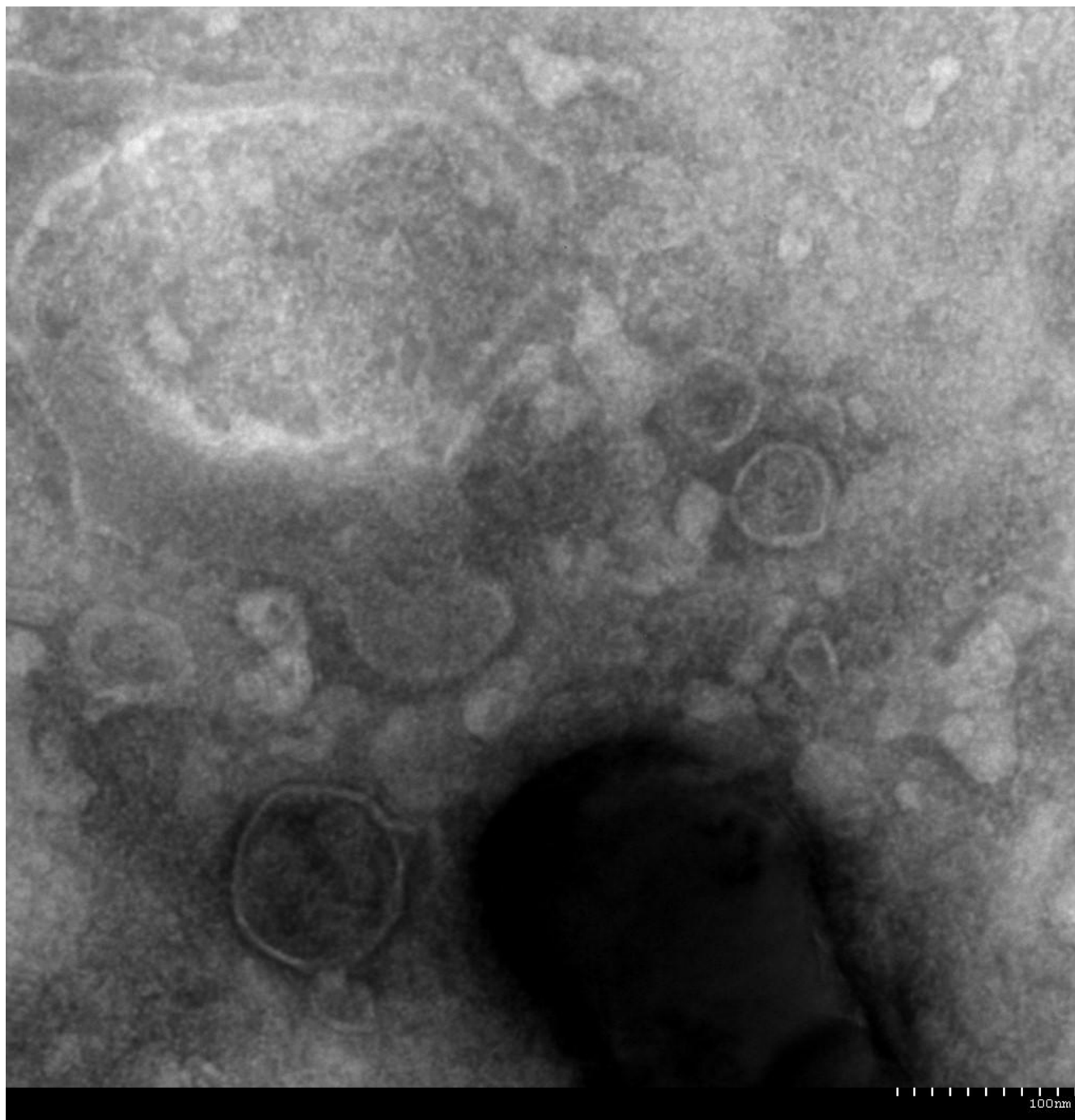

Supplement: Supplementary file 2 — Additional file 2: Figure S1. Wide-field image of EV morphology. [file 10020_2022_525_MOESM2_ESM.pdf]

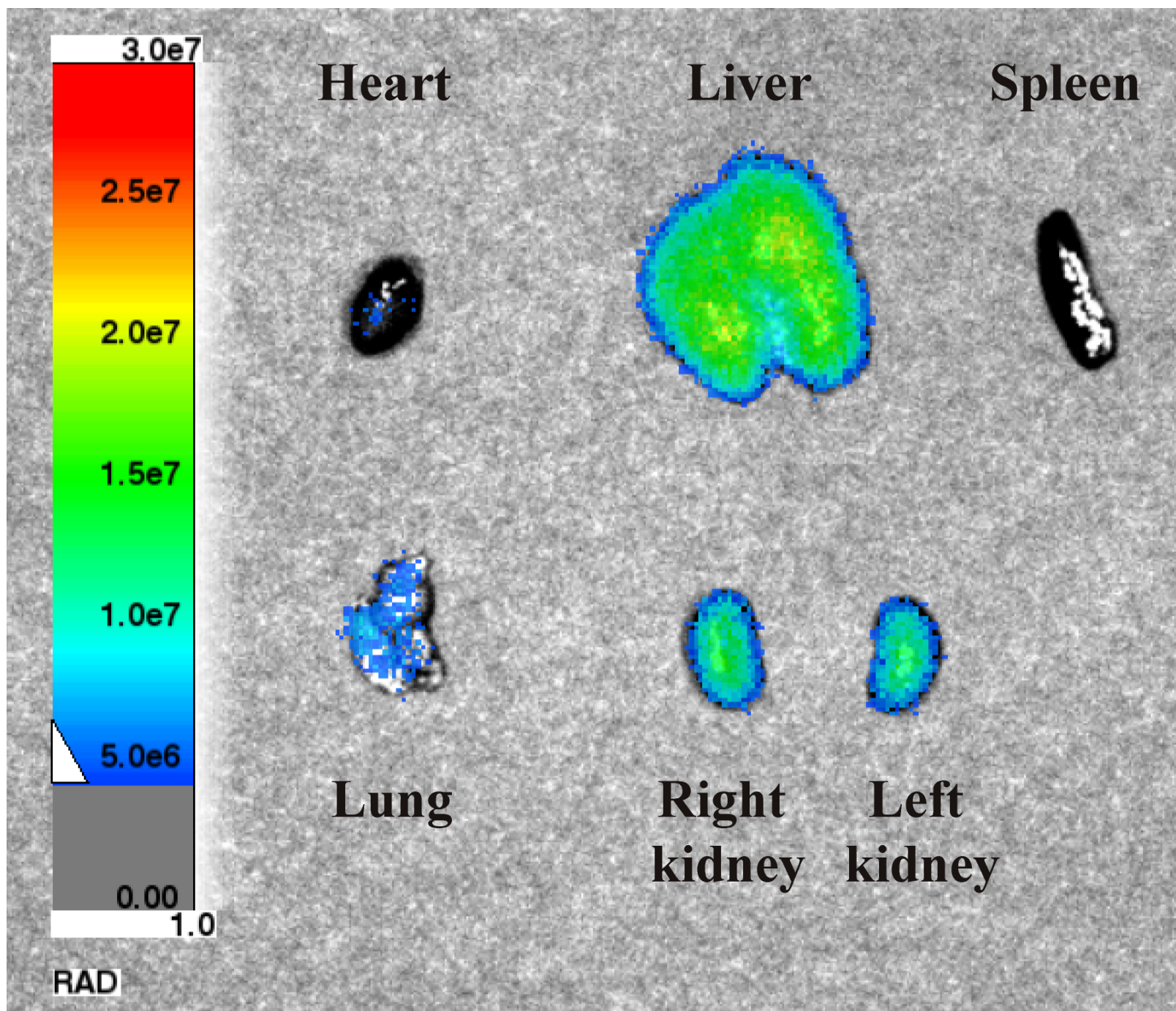

Supplement: Supplementary file 3 — Additional file 3: Figure S2. In vivo biodistribution of EVs in mice. Imaging of the fluorescence intensity in the mouse organs at 24 h after injection. [file 10020_2022_525_MOESM3_ESM.pdf]

A

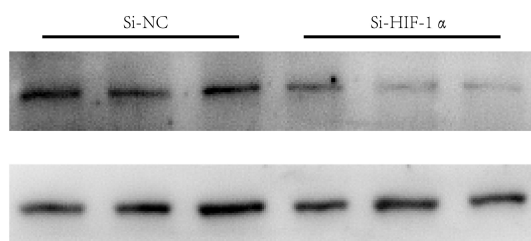

B

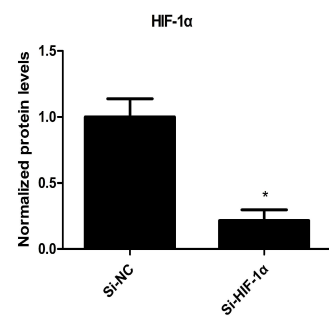

C

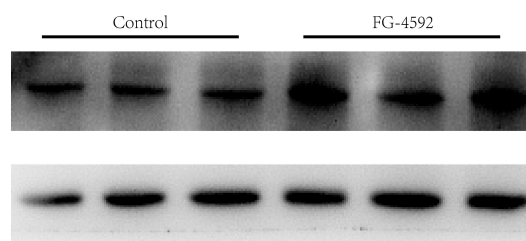

D

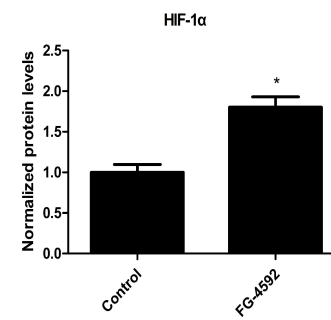

Supplement: Supplementary file 4 — Additional file 4: Figure S3. HIF-1α expression in macrophages. Macrophages were transfected with HIF-1α siRNA: (A, B) protein levels of HIF-1α (n = 3); *p < 0.05 vs. the Si-NC group. Macrophages were treated with FG-4592: (C, D) protein levels of HIF-1α (n = 3); *p < 0.05 vs. the control group. [file 10020_2022_525_MOESM4_ESM.pdf]
